# Identification of Myocardial Damage in Systemic Sclerosis: A Nuclear Cardiology Approach

**DOI:** 10.1155/2010/496509

**Published:** 2010-08-31

**Authors:** Kenichi Nakajima, Shinro Matsuo, Minoru Hasegawa, Seigo Kinuya, Kazuhiko Takehara

**Affiliations:** ^1^Department of Nuclear Medicine, Kanazawa University Hospital, Kanazawa 920-8641, Japan; ^2^Department of Dermatology, Kanazawa University Hospital, Kanazawa 920-8641, Japan

## Abstract

Myocardial involvement is an important prognostic factor in patients with systemic sclerosis, and early diagnosis and staging of the disease have been sought after. Since myocardial damage is characterized by connective tissue disease, including fibrosis and diffuse vascular lesions or microcirculation, nuclear myocardial perfusion imaging has been a promising option for evaluating myocardial damages in early stages. In addition to the conventional stress-rest perfusion imaging, the current use of quantitative electrocardiographic gated imaging has contributed to more precise evaluation of cardiac perfusion, ventricular wall motion, and diastolic function, all of which have enhanced diagnostic ability of evaluating myocardial dysfunction. Abnormal sympathetic imaging with Iodine-123 metaiodobenzylguanidine might be another option for identifying myocardial damage. This paper deals with approaches from nuclear cardiology to detect perfusion and functional abnormality as an early sign of myocardial involvement as well as possible prognostic values in patients with abnormal imaging results. The role of nuclear cardiology in the era of multiple imaging modalities is discussed.

## 1. Introduction

Systemic sclerosis (SSc) is a connective tissue disease characterized by diffuse vascular lesions and fibrosis, and it systemically involves various organs such as skin (scleroderma), heart, lung, kidney and gastrointestinal tracts [1–6]. Of these organ involvements, cardiac complications include arrhythmias, pericarditis, angina pectoris, congestive heart failure and sudden death. Autopsy findings demonstrated that myocardial fibrosis in SSc has been a common occurrence [1, 7]. Thus, it has become evident that early diagnosis and accurate staging of visceral involvement are fundamental for appropriate management and therapeutic approaches for SSc [8]. These approaches may provide a significant prognostic value to systemic sclerosis. Although the precise mechanism for pathogenesis and etiology is not the aim of this article, nuclear medicine approaches to SSc patients are presented in this paper. The mechanisms of cardiac dysfunction and insight that can be gained from nuclear imaging are discussed.

## 2. Subsets of SSc and Organ Involvements

SSc is usually classified into two subsets of diffuse and limited cutaneous types (dcSSc and lcSSc) [3]. The major findings of skin sclerosis and organ involvement are summarized in Table 1. Common manifestations of organ involvement in dcSSc include interstitial lung disease, renal failure, diffuse gastrointestinal disease, and myocardial involvement. It has been found that cardiac involvement is more common in patients with dcSSc, and one of the least predictable of the visceral involvements during the clinical course of dcSSc. However, even in the lcSSc subset, ischemic response has been detected in 64% of the patients using thallium-201 (^201^Tl) myocardial perfusion imaging [9]. A research group database from the EULAR scleroderma trials showed that scleroderma subsets (lcSSc and dcSSc types), autoantibody status and age at onset of Raynaud's phenomenon were found to be independently associated with the prevalence of organ manifestations [10]. It was also important to separate patients into two SSc subsets for the purpose of survival analysis. Poorer prognosis was associated with the dcSSc type, positive antitopoisomerase I antibody and negative anticentromere antibody in the long-term followup [11–13].

## 3. Nuclear Cardiology Studies for Cardiac Involvement in SSc and Pathophysiological Bases

### 3.1. Myocardial Perfusion Imaging and Underlying Pathophysiology

In nuclear cardiology, myocardial perfusion imaging has been used extensively for evaluating coronary artery disease, which includes diagnosis of ischemic heart disease, physiological assessment of known coronary stenosis, viability assessment after acute coronary syndrome, reevaluation after coronary intervention, and risk stratification for future cardiac events [14]. The diagnostic sensitivity of coronary artery disease is approximately 80%–90%, and its specificity is around 70%–80%. The advent of electrocardiography (ECG) gated perfusion imaging has further enhanced diagnostic accuracy by simultaneously evaluating myocardial ischemia and functional abnormality [15]. 

In more than three decades of history of nuclear medicine in cardiology, an early finding of myocardial perfusion abnormality in SSc was documented in 1984 by planar ^201^Tl perfusion imaging with circumferential profile analysis that added quantitative support [16, 17]. Coronary angiography was normal in those patients. A reduced coronary flow reserve has also been documented without coronary stenosis. A subsequent study using cold-stress showed transient myocardial perfusion defects as visualized by ^201^Tl [18]. The authors suggested that cold exposure in SSc patients might elicit transient reflex coronary vasoconstriction resulting in reversible myocardial ischemia and dysfunction. Using cold stress and dipyridamole stress, half of the patients with long-standing Raynaud's phenomenon presented ischemic ^201^Tl defects [19]. It is noteworthy that scleroderma patients with a normal dipyridamole test demonstrated cold-induced transient myocardial ischemia. Thus, primary involvement is not major coronary artery stenosis in SSc, but the target of perfusion abnormality is related to microcirculation. Despite the potential differences in imaging targets, nuclear medicine studies with ^201^Tl and Technetium-99m (^99m^Tc)-labeled radiopharmaceuticals have shown that either stress-induced ischemia or persistent perfusion defects occur in SSc patients [18–24]. After the advent of single-photon emission computed tomography (SPECT), the detectability of small perfusion defects was enhanced. A study with ^201^Tl SPECT in patients with SSc and systemic lupus erythematosus showed a high incidence of (82%) of abnormal findings by ^201^Tl SPECT [25]. The authors used quantitative analysis with a polar map and a 17-segment model, and found reverse redistribution finding in patients with collagen diseases. 

Based on a pathophysiological viewpoint, abnormalities of microcirculation seemed to play an important role leading to myocardial damages [16]. Focal myocardial lesions ranging from contraction band necrosis to fibrosis and reversible vasospastic abnormality in small coronary arteries have been found to be a key mechanism in myocardial dysfunction. Patchy scarring and focal necrosis unassociated with coronary artery disease were observed, and pathologic changes were sometimes associated with fibrous pericarditis and a cardiac conduction system [26]. Myocardial perfusion abnormality may be related to scattered or diffuse fibrosis and contraction band necrosis due to vasospasm or repeated focal ischemia [7, 27, 28]. When the severity of disease advances, perfusion defects may become larger or might be detected by an exercise stress protocol. However, in the early stage, the abnormality might not be evident based on conventional exercise-stress protocol. Vasodilator imaging, currently using dipyridamole, adenosine triphosphate, and adenosine, might be useful for detecting ischemia. However, pharmacological vasodilator stress would be more appropriate for detecting ischemia by atherosclerotic coronary stenosis, rather than by vasospastic microcirculation abnormality. Conversely, dipyridamole significantly improved resting ^201^Tl myocardial perfusion: the mean number of segments with perfusion defects decreased from resting condition after dipyridamole [29]. The same group also revealed short-term improvement in ^201^Tl myocardial perfusion with nifedipine in patients with progressive systemic sclerosis [23]. In contrast, fixed perfusion defect, either scattered or segmental, reflects myocardial fibrosis, and stress-induced ischemia reflects vasospasm of small coronary arteries or coronary stenosis. Both ischemia and fibrosis coexist in an individual heart, and the induced ischemia may be reversible and potentially treated by medications.

### 3.2. Left Ventricular Dysfunction in SSc

Resting left ventricular ejection fraction (EF) and its response to exercise also appeared to be unrelated to the findings on ^201^Tl scanning, except for subtle abnormality [17]. However, ^201^Tl perfusion defects appeared to be related to left ventricular function, which showed that patients with perfusion defect had scores above the median value. In contrast, several patients with diffuse scleroderma had prominently abnormal EF at rest with further prominent deterioration during exercise [16]. Thus, a baseline decrease in left ventricular contractility may be related to resting perfusion defects that reflect myocardial fibrosis.

In a Japanese population, we performed a stress ^99m^Tc methoxy-isobutyl-isonitrile (MIBI) study with ECG-gating of 16 frames per cardiac cycle in 34 patients with SSc [30]. Compared with Western studies as described above, in contrast, only slight segmental defect and/or stress-induced ischemia were observed by perfusion SPECT (Table 2). We found a significant relationship between diastolic abnormality and modified Rodnan total skin thickness score (MRSS) [5, 31]. A decreased resting EF of less than 55% was found in no patients in the low-MRSS group and in two patients in the high-MRSS group. However, diastolic dysfunction was observed in the high-MRSS group. The time to peak filling rate differed significantly among the control, low-MRSS and high-MRSS groups. Impaired relaxation and diastolic asynchrony in SSc were also reported using radionuclide angiography and echocardiography [32, 33]. 

Diastolic dysfunction as detected by either gated myocardial perfusion imaging or radionuclide ventriculography was not specific to the findings of SSc. Diastolic dysfunction may have occurred even without any systolic dysfunction in patients with ischemic heart disease, hypertrophic cardiomyopathy, hypertensive heart diseases, and secondary cardiomyopathies [34–37]. In patients with the clinical syndrome of heart failure, determination of the presence and severity of diastolic dysfunction is increasingly important [14]. Complicated factors such as myocardial stiffness, wall elasticity, compliance, incomplete relaxation, and ventricular pressure may be involved in diastolic dysfunction, and a similar mechanism may also be at work in SSc. We postulated that diastolic function was an early sign of cardiac involvement, since it appeared even in patients with neither perfusion abnormality nor systolic dysfunction.

Thus, we postulate that myocardial perfusion SPECT abnormality is correlated to the severity of cardiac dysfunction. In mild dysfunctional patients, even when left ventricular contractility is normal, diastolic dysfunction may be related in some cases. A stress myocardial perfusion scan might show reversible perfusion defect in patients with mild to moderate severity. In patients with more advanced severe dysfunction, fixed perfusion defects and regional wall motion abnormality would occur accompanied by global decrease in EF. However, whether or not the slight degree of perfusion abnormality and diastolic dysfunction would result in further deterioration of cardiac function should be investigated in a long-term follow-up study.

The reason for the relatively low incidence of perfusion abnormality in our study might be explained by the selection bias of our study population, which might not have included the severer type of SSc. As for another possible reason, it might have been caused by differences in the sex and race of the patients, since ethnic differences have also been demonstrated in a Japanese population [38, 39].

### 3.3. Additional Information by Quantitative Gated SPECT Analysis

**Figure 1:**
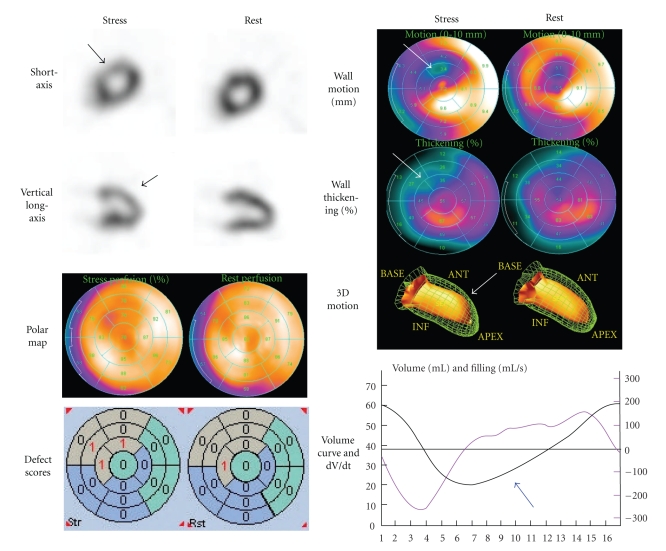
A patient with SSc showing slight anteroseptal ischemia. Quantitative analyses of perfusion, defect scores, wall motion and thickening showed significant abnormality, which supported abnormality in this region (arrows). Diastolic dysfunction was observed even at resting condition as shown by the blue arrow.

Our experience with SSc patients has shown that large perfusion defects and induced ischemia do not seem to be so common as described previously. Instead, small regional abnormality has become evident by the help of quantitative analysis. Current nuclear cardiology technology uses sophisticated computer-aided diagnosis for quantification. As an example, a patient with SSc is shown in Figure 1.

A 66-year old male patient diagnosed with dcSSc was referred to our department for nuclear cardiology for the purpose of evaluating possible cardiac involvement. His onset of disease was Raynaud's phenomenon 5 years ago prior to his visit. Scleroderma progressed since 2 years ago extending from hands, forearms, anterior chest to abdominal skin, and the MRSS indicated moderate severity (score 25 of 51). Significant pulmonary fibrosis was observed by X-ray computed tomography (CT). Myocardial perfusion SPECT was performed with ^99m^Tc MIBI using stress-rest protocol. During exercise, only a slight decrease in perfusion was observed in the apical anteroseptal walls. A polar map display, with a 17-segment model overlaid, showed a localized decrease in perfusion in the anteroseptal region. The summed stress score and rest score namely, semi-quantitative defect scores during exercise and at rest were 3 and 1, which was judged as only a slight abnormality. In the polar map, wall motion and systolic thickening were apparently reduced in the anteroseptal region, particularly after exercise. A three-dimensional ventricular contour display showed apical anterior hypokinesis. A volume curve by gated SPECT showed characteristics of diastolic dysfunction. The time to peak filling rate (TPFR) as defined from the time points from end-systole to peak filling rate was prolonged to 352 msec (normal value for Japanese population: 159 ± 26 msec (SD)). The early diastolic parameter of 1/3 mean filling rate was 0.94 /sec (normal value: 1.68 ± 0.30 /sec)) [40]. Instead of simply judging positive or negative perfusion defects, we were able to definitely diagnose the abnormality in the SPECT study by integrating various quantitative parameters.

### 3.4. Possible Options of Radiopharmaceuticals

**Figure 2:**
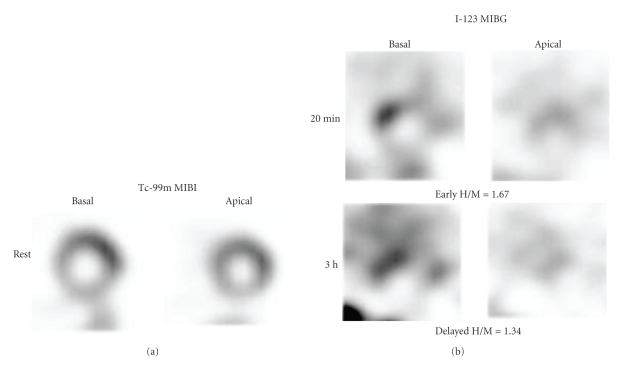
A patient with diffuse cutaneous type with MRSS 21, showing decreased MIBG activity and rapid washout rate (33%). ^123^I-MIBG distribution showed marked heterogeneity in both early and delayed short-axis images. Resting perfusion was normal by ^99m^Tc-MIBI SPECT. Adapted from [41].

Myocardial perfusion imaging is fundamental for evaluating myocardial damage. In addition, patients with SSc have a high frequency of diastolic dysfunction and sympathetic abnormality as shown by ^123^I-metaiodobenzylguanidine (MIBG) studies [41, 42]. ^123^I-MIBG is known as an analogue of norepinephrine and shares its metabolic pathways with catecholamine uptake and excretion. Sympathetic abnormality has been detected by decreased uptake and rapid clearance from the heart. Initial experiences of MIBG support its use in ischemic heart diseases, typically shown as regional denervation of the heart in acute coronary syndromes and cardiomyopathies. Currently, ^123^I-MIBG has been used for patients with heart failure as well as neurological disorders involving Lewy-body diseases [43–45]. It has been understood that MIBG uptake reflects sympathetic neuronal activity or norepinephrine contents, and increased sympathetic activity drives results in rapid MIBG clearance. Figure 2 shows a patient with normal myocardial perfusion associated with severe decrease in MIBG activity. In this patient, early and delayed heart-to-mediastinum average count ratio (H/M) was 1.67 and 1.34, respectively (normal values: 2.39 ± 0.21 and 2.49 ± 0.25, resp., [40]). MIBG washout rate also increased to 33% (normal value was <20% [46]). In concordance with the MIBG abnormalities, some studies have indicated that autonomic dysfunction was extremely common in patients with SSc. It was characterized by parasympathetic impairment and marked sympathetic overactivity, particularly in the early stage [47, 48]. However, a cause and effect relationship between autonomic derangement and repeated myocardial damage has not been clearly identified. 

The use of MIBG imaging has also been validated by a large prognostic multicenter trial in patients with heart failure of New York Hear Association (NYHA) functional class II/III and LVEF of 35% or less [49]. The two-year event rate was 15% for H/M ≥1.60 and 37% for H/M <1.60. The authors found that ^123^I-MIBG provided additional discrimination in analyses of interactions between B-type natriuretic peptide, LVEF and H/M ratio. Although pathophysiological evidences of heart failure in the study could not be readily applicable to SSc patients, the MIBG imaging may provide new insights into the nature and prognosis of myocardial damage in SSc.

An article reported a case of systemic sclerosis with a subacute episode of myocardial disease assessed by ^111^In-antimyosin antibody, which was a marker of the myocardial damage or necrosis [50]. However, antimyosin antibody was considered to accumulate in ongoing myocardial injury typically caused by ischemia or infarction, and thus it may not be applicable for an early stage of systemic sclerosis.

## 4. Prognostic Value of Nuclear Cardiology in Systemic Sclerosis

Large-scale prospective cohort studies have been performed extensively in the field of coronary artery disease, although such prognostic studies are still limited in patients with SSc. However, it has been recognized that cardiopulmonary involvement has been considered to be a poor prognostic factor since renal involvement is no longer a major cause of death in SSc, [7, 27, 51]. Steen and Medsger demonstrated that when natural history and timing of severe organ involvements were analyzed in the dcSSc, the 9-year cumulative survival rate of all patients with severe organ involvement was 38%, compared with 72% in patients without such involvement [52]. Long-term prognosis was found to be poor in patients with ^201^Tl perfusion defect. A study followed up 48 patients who underwent a perfusion scan in the 1980s. Notably, the survival information over the last 10 years revealed that the size of the initial defect was the best predictor of later adverse events compared with other disease-related features. These kinds of long-term prognostic study, which has been accumulating in the field of coronary artery disease, should also be conducted in SSc patients both retrospectively and prospectively.

## 5. Practical Approaches to Cardiac Evaluation in SSc

**Figure 3:**
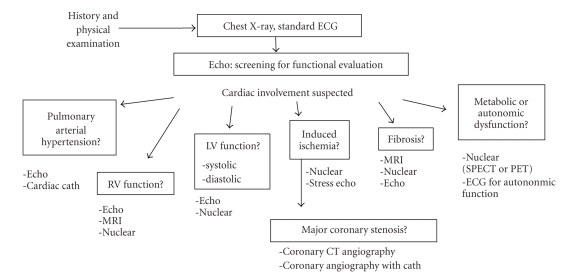
Possible roles of cardiac imaging modalities for diagnostic work-ups and followup in SSc. Abbreviations: ECG, electrocardiography; Cath., catheterization; MRI, magnetic resonance imaging; CT, computed tomography; SPECT, single-photon emission computed tomography; PET, positron emission tomography.

Although we have focused on nuclear cardiology methods, echocardiography remains the mainstream tool for heart evaluation in SSc patients [6, 27, 53]. Diagnostic work-ups in patients with SSc are summarized in Figure 3 from the viewpoint of cardiac imaging. The first step for the diagnosis starts by careful evaluation of the history and symptoms, followed by the physical examination including skin sclerosis and possible organ involvements. The baseline 12-lead ECG and chest X-ray examination are standard procedures for screening. When considering the possibility of cardiac involvement, echocardiography, coupled with Doppler if possible, would be the first-line methodology for cardiac functional evaluation. This approach would show the presence of pericardial effusion, right ventricular involvement as well as left ventricular systolic and diastolic function. 

When cardiac abnormalities are suspected by the initial screening tests, the next step would be diverse, depending on the clinical problems in the individual patient. In patients with pulmonary arterial hypertension (PAH), guidelines from both the American College of Chest Physicians (ACCP) and European Society for PAH recommended chest-X-ray, ECG and Doppler echocardiography as evidence-based approaches [54, 55]. The prevalence of pulmonary hypertension associated with scleroderma ranged from 4.9% to 38% as written in the ACCP guidelines. In addition, cardiac catheterization for the assessment of pulmonary hypertension might still be required in some patients who can benefit from intervention. The appropriate evaluation of PAH has practical values for guiding medical treatment, as four sets of recommendations were formulated for SSc-related PAH by EULAR scleroderma trials and their research group [56]. The ACCP also revised the treatment algorithm by taking into account recent developments in therapy [57]. Nuclear cardiology has no validated role for this purpose, although lung ventilation/perfusion may be evaluated in chronic thromboembolic pulmonary hypertension, and metabolism in the right ventricular overload [55, 58, 59]. Right ventricular function may also be evaluated by echocardiography or gated blood-pool study [60]. However, when a major concern of the patient problem involves induced myocardial ischemia, a stress nuclear study with vasodilator or vasoconstrictor stressors would be the best option for guiding management. If stress-induced large perfusion defects were found in the stress study, coronary work-ups with CT angiography using contrast media would be selected. Coronary evaluation may not be indicated when the perfusion defect is relatively small and microvascular origin is more likely. When myocardial fibrosis is suspected based on the ventricular contractility, regional wall motion or diastolic function, the severity might be evaluated by a nuclear study. Recently, using delayed enhanced magnetic resonance imaging (MRI), several studies successfully identified myocardial fibrosis in a significant percentage of patients with SSc [27, 61, 62]. The SPECT or positron emission tomography (PET) may identify larger defects and/or associated ischemia. No comparative study for their effectiveness has been performed to date.

According to our study, it is apparent that all patients diagnosed with SSc are not indicated for a SPECT study. Based on our study population, it is noteworthy that patients with the lcSSc type or MRSS less than 10 showed neither significant perfusion defect nor ventricular dysfunction (Table 2). Therefore, we tentatively recommend a myocardial perfusion study in patients with dcSSc type and/or MRSS greater than 10, when the patient is suspected of having cardiac abnormality [30, 41]. Belloli et al. studied potential risk factors for microvascular involvement and found that perfusion defects were related to skin scores, digital ulcers, and esophageal involvement, and these patients might warrant screening for myocardial involvement [24]. Thus, patients with SSc who have a history of chest symptoms, conduction abnormality or arrhythmia on ECG, and wall motion abnormality and those who have findings of multiple organ complications would be potential candidates for myocardial SPECT imaging. 

The best diagnostic approach or decision tree for cardiac evaluation has not been defined yet, although chest X-ray and echocardiography would be the first-step imaging method readily available in any hospital. While several possible diagnostic approaches have been proposed, some of them might have practical values for diagnosis, and others may provide pathophysiological insight or prognostic information. The determination of the appropriate role of imaging is of great concern in the era of multimodality cardiac imaging.

## 6. Conclusion

Myocardial involvement as part of diffuse organ fibrosis and vascular changes is commonly observed in SSc. Even when severe fibrosis as evidenced by myocardial perfusion defects is not observed, perfusion reserves or functional abnormality might be detected as an earlier sign of myocardial damage. The aid of computer-assisted diagnosis will enhance the diagnostic ability for identifying abnormalities. The relationship of nuclear imaging and prognosis should be evaluated to confirm the role of functional imaging for patients with SSc. Finally, the roles or effectiveness of various current imaging modalities remain to be defined.

## Figures and Tables

**Table 1:** SSc subsets and organ involvements.

	Diffuse cutaneous SSc	Limited cutaneous SSc
Skin sclerosis	Truncal and acral skin involvement	Limited to hands, feet, face, and forearms, or absent
Organ involvement	Early and significant incidence of interstitial lung disease, oliguric renal failure, diffuse gastrointestinal disease, and myocardial involvement	Significant late incidence of pulmonary hypertension, trigeminal neuralgia, calcinosis, and teleangiectasia
Antibodies	Anti-DNA topoisomerase I antibodies	Anticentromere antibodies

**Table 2:** Comparison of low and high skin thickness score in patients and control subjects.

	Control	Low MRSS	High MRSS	*P*
		(<10)	(≥10)	
N	16	16	18	
Age (years)	50 ± 12	56 ± 10	55 ± 15	n.s.
Male : female	2 : 14	2 : 14	1 : 17	n.s.
MRSS	—	4.0 ± 2.5	19.2 ± 6.7	<.0001

*Myocardial perfusion imaging*				
Induced ischemia	0	2	1	n.s.
Resting hypoperfusion	0	2	3	n.s.

*Gated SPECT*				
Heart rate (/min)	65 ± 7	68 ± 11	71 ± 8	n.s.
EF (%)	68 ± 9	73 ± 9	71 ± 12	n.s.
EF <55%	0 (0%)	0 (0%)	2 (11%)	n.s.
PFR (/sec)	2.46 ± 0.45	2.76 ± 0.44	2.74 ± 0.53	n.s.
1/3 MFR (/sec)	1.52 ± 0.25	1.57 ± 0.31	1.25 ± 0.42	.017
TPFR (msec)	166 ± 22	168 ± 38	216 ± 82	.015
TPFR/RR	0.18 ± 0.02	0.19 ± 0.04	0.26 ± 0.09	.002

Adapted from the results of [30]

MRSS, modified Rodnan total skin thickness score;

EF, ejection fraction; PFR, peak filling rate; 1/3 MFR, one-third mean filling rate;

TPFR, time to PFR; TPFR/RR, TPFR divided by RR interval.
